# Phonon imaging in 3D with a fibre probe

**DOI:** 10.1038/s41377-021-00532-7

**Published:** 2021-04-27

**Authors:** Salvatore La Cavera, Fernando Pérez-Cota, Richard J. Smith, Matt Clark

**Affiliations:** Optics and Photonics Group, Faculty of Engineering, University of Nottingham, University Park, Nottingham, NG7 2RD UK

**Keywords:** Imaging and sensing, Fibre optics and optical communications

## Abstract

We show for the first time that a single ultrasonic imaging fibre is capable of simultaneously accessing 3D spatial information and mechanical properties from microscopic objects. The novel measurement system consists of two ultrafast lasers that excite and detect high-frequency ultrasound from a nano-transducer that was fabricated onto the tip of a single-mode optical fibre. A signal processing technique was also developed to extract nanometric in-depth spatial measurements from GHz frequency acoustic waves, while still allowing Brillouin spectroscopy in the frequency domain. Label-free and non-contact imaging performance was demonstrated on various polymer microstructures. This singular device is equipped with optical lateral resolution, 2.5 μm, and a depth-profiling precision of 45 nm provided by acoustics. The endoscopic potential for this device is exhibited by extrapolating the single fibre to tens of thousands of fibres in an imaging bundle. Such a device catalyses future phonon endomicroscopy technology that brings the prospect of label-free in vivo histology within reach.

## Introduction

In this report we present the first optical fibre-based ultrasonic imaging tool capable of resolving biological cell-sized objects. The device simultaneously accesses topographic and material information from microscopic objects. This is accomplished using a novel signal processing protocol that renders a spatial measurement from the amplitude decay signature of the time-of-flight of a GHz frequency acoustic wave. Proof of concept profilometry and spectroscopy is carried out on a Petri dish and polymer microstructures. It is also demonstrated that the technology is compatible with both single-mode optical fibre and the multi-mode channels of an imaging bundle.

High resolution is achieved by a unique combination of optical lateral resolution and acoustic axial precision. Lateral resolution is set by the mode field diameter of the optical fibre; axial precision is enabled by the phonon wavelength and temporal resolution of the signal processing. Furthermore, the acoustic time-of-flight can be analysed to obtain viscoelastic information by measuring the sound velocity and attenuation of the wave. Due to the partial transparency of the ultrasonic sensor, the device in bundle-format can still be used for brightfield or fluorescence imaging.

The optical fibre and imaging bundle implementations of this technology hold promise for integration into standard endoscopy and endomicroscopy equipment. Sub-cellular resolution provides an opportunity to perform three-dimensional (3D) in vivo histology without the fluorescent labels required by similarly resolved endoscopic techniques. The availability of additional elastic information could also introduce a novel histological metric with which to characterise disease at the point of care. Beyond clinical healthcare, the fields of tissue engineering and precision manufacturing could also utilise this high-resolution tool for superficial diagnostics.

Sub-cellular resolution in optical endoscopy has been most readily achieved by a single optical fibre, which is not limited by the core-to-core spacing of a fibre bundle^1^. In general, a single optical fibre can be used to form images by the following means: (1) scanning the distal end of the fibre from point-to-point in the object plane or (2) using a multi-mode fibre to encode spatio-angular information across the range of core modes^2,3^. Lacking this latter capability, single-mode optical fibres are typically used in distal scanning^1^ or spatially dispersive^4^ configurations, and have provided breakthroughs in confocal endomicroscopy^2,5,6^ and endoscopic optical coherence tomography^7^. On the other hand, a single multi-mode fibre is less dependent on scanning since each mode within the fibre acts as a pixel; the caveat is that mode dispersion scrambles the image information and must be empirically compensated^8^. Once unscrambled, the multi-mode fibre empowers lensless endoscopy with qualities such as high numerical aperture (NA)^9^, wide field^10^, 3D imaging^11^ and even super-resolution^12^.

Practically speaking, there are certain limits to the utility of purely optical endoscopy techniques. For example, cellular tissue often exhibits poor optical contrast and specificity, which is typically mitigated within sub-cellular resolution endomicroscopy and endocytoscopy by staining the tissue with fluorescent labels^2,3,5,6,13^. However, acoustics natively offers a pathway to high contrast imaging within biological media, as demonstrated by an extensive history of imaging modalities^14–19^.

Compared with optical techniques, acoustics has long been hampered by a lack of resolution which can be attributed to the extreme measures required to reduce the acoustic wavelength, e.g., (1) miniaturising piezoelectric transducer systems and (2) debilitating acoustic attenuation in liquids at high frequencies. Consequently, the same development arc for cellular resolution optical endoscopy has not been duplicated in acoustics. Following the advent of the scanning acoustic microscope in 1974 (ref. ^20^), the most pragmatic breakthroughs in high-resolution acoustics have been provided by opto-acoustics, i.e., the optical detection of acoustic phenomena^14–16,21–25^. Among these techniques, picosecond ultrasonics (PU)^26^ and Brillouin scattering^27^ are of particular interest as they offer picosecond temporal resolution and direct read-out of viscoelastic properties (respectively) with optical lateral resolution. Time-resolved Brillouin scattering synergises these concepts and has enabled 3D elastography of biological cells with sub-optical wavelength phonons^28,29^.

Despite significant progress in these fields, the maximum available resolution of all-optical ultrasonic 3D-imaging fibres is on the order of ~40 μm^30^, and therefore the reality of cellular resolution acoustic endoscopy remains elusive. Presented here is the first optical fibre-imaging tool for acoustic microscopy. Similar to other state-of-the-art optical fibre-imaging techniques, this 125 μm diameter single-mode *phonon probe* is arranged in a distal scanning, front-view, and reflection-mode configuration. What follows are principles of operation for the device, results demonstrating label-free parallel profilometry and spectroscopy, and the related performance metrics. Furthermore, it is demonstrated that the single fibre can be extrapolated to a wide-field imaging bundle which enables a new class of phonon endoscopes.

## Results

### Working principle

**Fig. 1:**
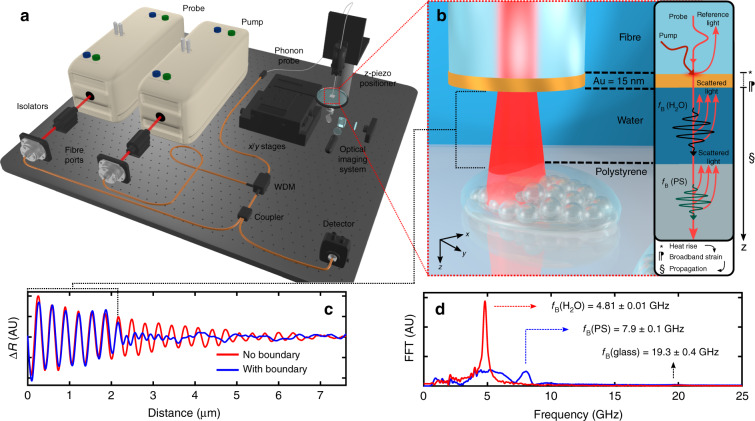
Design, mechanism, and signal response of the phonon probe. **a** Experimental layout of the phonon probe system (further details in ‘Materials and methods’). **b** The phonon probe approaches a polystyrene (PS) object immersed in water. (inset) Absorption of a pump pulse heats the transducer which relaxes in the form of broadband strain (propagating away from the fibre-tip). A probe pulse partially reflects at the transducer interface (reference beam), Brillouin scatters with the *f*_B_(H_2_O) mode in water (black waveform), and later in signal time (a subsequent probe pulse) Brillouin scatters with the *f*_B_(PS) mode (green waveform). Co-propagating reference and scattered light (now counter-propagating in the system) interfere at the detector, producing a modulation in reflectivity (Δ*R*). **c** The transition in materials is observed in the time/spatial domain for the blue TRBS signal (water for Δ*R*(*z* < 2 μm) and PS for Δ*R*(*z* > 2 μm)), or is unobservable if there is no object impeding the measurement volume (red curve). **d** Fast Fourier transform (FFT) of the TRBS signals reveals the constituent Brillouin frequencies of the respective materials

The basic mechanism of the phonon probe is to inject coherent acoustic phonons (CAPs) into a specimen, and detect its vibrational response through the optical effect of Brillouin scattering. For acoustic excitation, the phonon probe utilises the principles of PU: a metallic thin-film generates GHz–THz bandwidth CAPs through the photoacoustic effect^26^. In practice, this is realised by fabricating a partially transparent gold nano-transducer onto the distal end-face of a single-mode optical fibre (Fig. 1b), and periodically exciting the transducer with short pump pulses within a pump-probe spectroscopy system (see Fig. 1a and ‘Materials and methods’ for more details).

Detection is achieved by illuminating the CAP-excited region with a series of short probe pulses. Each optical pulse exchanges energy with the CAP, through the effect of Brillouin scattering, and the optical frequency of the pulse shifts according to the phonon frequency, i.e. the Brillouin frequency:

1 $$f_{\mathrm B} = \frac{{2nv}}{{\lambda _{\mathrm {probe}}}}$$

(for normal probe light incidence), where *n* is the refractive index of the surrounding medium, *λ*_probe_ is the optical wavelength, and *v* is the sound velocity in the medium. By progressively delaying the timing between pump (generation) and probe (detection) pulses, each successive probe pulse Brillouin scatters with a later segment of the phonon path length. Back-scattered probe light (‘scattered light,’ Fig. 1b) then re-couples into the optical fibre core and counter-propagates in the optical system. At the detector, this pulse train of frequency shifted light interferes with an unshifted probe reference beam, provided by a partial reflection from the gold layer (‘reference light,’ Fig. 1b). The interference pattern (in time) generated by this process contains a beat frequency: *f*_B_. In other words, the detector records the time-of-flight of the CAP (Δ*R* in Fig. 1c) with frequency *f*_B_; the temporal extent of this signal (*t*) can be reconciled with axial distance (*z*) using the sound velocity in the medium: *z* = *vt*. The above process is time-resolved Brillouin scattering (TRBS)^31,32^, first implemented in optical fibre format by the phonon probe^33^.

Measuring the Brillouin frequency, e.g., by transforming the time domain signal into the frequency domain (Fig. 1d), allows one to quantify the sound velocity, provided that the refractive index is known. Additionally, the rate at which the specimen dissipates acoustic energy can be measured by tracking the amplitude decay (attenuation coefficient, *α*) of the signal with time. However, measurement of the latter depends on a number of competing effects, such as opto-acoustic defocus^34^ and mechanical heterogeneity^35^, which must be considered before isolating *α*. Together, sound velocity and attenuation measurements provide an opportunity to quantify viscoelasticity in the form of the complex longitudinal modulus: *M* = *M’* + *iM″* = *v*^2^*ρ* + *i*4*πf*_B_*αρ*, provided that the mass density (*ρ*) of the measurement volume is known^35^.

A unique aspect of TRBS is that viscoelastic information in the axial direction can be measured using the phonon wavelength (*λ*_phonon_), which at GHz frequencies (Brillouin range) is shorter than the optical probe wavelength (due to the Bragg condition: *λ*_phonon_ = *λ*_probe_*/2n*) which governs the Brillouin scattering interaction. This is in contrast with spectrometer-based (non-time resolved) confocal Brillouin imaging techniques^14^ that utilise the same phase matching criterion provided by the Bragg condition, yet are limited by the optical depth of focus for in-depth sampling.

In this paper, we propose a method for applying the sub-optical axial resolution of the phonon probe to resolve the nanometric topography of an object: picosecond ultrasonic profilometry (PUP). If the phonon probe is immersed in a couplant medium (water, *f*_B_(H_2_O)) and brought sufficiently close to a photoelastic object (polystyrene (PS), *f*_B_(PS)), a transition in Brillouin frequency^36,37^ will be observed in the TRBS signal (blue curve in Fig. 1c). This scenario presents two opportunities. (1) Quantifying elastic properties of the object, e.g., by analysing the Brillouin spectrum (blue curve, Fig. 1d). (2) Measuring the proximity between the fibre-tip and the object by finding the temporal position of the frequency transition. This is enabled by measuring the average Brillouin frequency in the couplant medium (red peak in Fig. 1d), and therefore the average sound velocity in the couplant (solving Eq. (1) for *v*), which converts the time position of the transition into an axial spatial position for a single pixel. We reiterate that the measurement of the local sound velocity requires a priori knowledge of the refractive index in the couplant medium. Using room temperature water as a couplant, we make the assumption that the medium is homogeneous, and has refractive index *n*_water_ = 1.33. By scanning this process in two-dimensions, it is possible to perform parallel 3D topographical mapping and spatially resolved elastography, all with sub-optical axial resolution, optical lateral resolution, true non-contact (between transducer and object), and label-free operation.

### In-depth measurement process

**Fig. 2:**
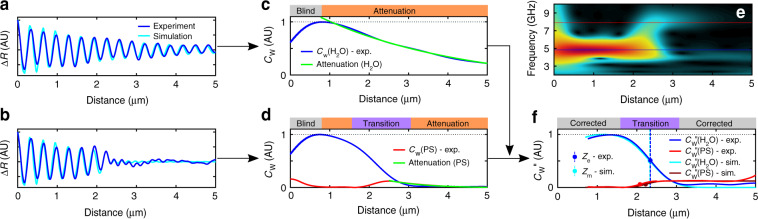
Proximity measurement protocol for the phonon probe. **a** A reference signal, when there is no boundary, permits the acoustic attenuation rate of the couplant to be characterised through wavelet analysis (green fit in **c**). **b** When there exists a boundary, a transition in Brillouin frequency occurs, accompanied by a sharp decay in the amplitude at the carrier frequency (blue in **d**) and a rise in the amplitude at the Brillouin frequency of the second material (red in **d** with green attenuation fit). **e** Spectrograph of the signal in **b** showing the constituent acoustic modes: blue line for ~5 GHz (water), and red line for ~8 GHz (PS). **f** The attenuation rate of the carrier frequency normalises the boundary decay (*C*_*w*_″, see Eq. (3)); markers indicate the position of 50% amplitude between the absolute minimum and maximum amplitudes of the response, thus providing a proximity measurement from the tip (*z*_e_ for experiment and *z*_m_ for model)

To define and characterise the in-depth measurement process, a PS Petri dish filled with water was used as a test specimen. Initially a reference TRBS signal was recorded with the fibre-tip positioned distantly from the surface of the Petri dish, yet still in the water couplant (blue curve in Fig. 2a). The purpose of this measurement is to define the state of *no object* for the phonon probe. To classify the existence of an object, the fibre-tip was moved toward the surface of the Petri dish in discrete steps until a change in the reference signature was observed (e.g. transitioning from Fig. 2a, b). TRBS signals were recorded roughly every 3 s (per step), and the process was completed within the span of minutes; in the future this process can be accelerated by automating the fibre approach, similar to the stylus engagement process in atomic force microscopy. Figure 2b shows the response of the phonon probe when the PS surface enters the measurement window: an initial 4.81 ± 0.01 GHz low-frequency oscillation emanating from the tip of the transducer (*z* = 0) which sharply transitions into a 7.9 ± 0.1 GHz high-frequency oscillation at *z* ≈ 2 μm. These Brillouin frequencies are characteristic to water and PS at the optical probe wavelength of 850 nm, and agree with values found in literature.

Now consider that the position of the frequency transition in Fig. 2b is a proximity measurement between the surface of the Petri dish and the fibre-tip which can be extracted through time-frequency analysis. We used the continuous wavelet transform (CWT) for time-frequency analysis, which has been utilised previously in PU to localise interfaces by identifying the positions of acoustic echoes^38^ or transitions in acoustic amplitude at the Brillouin frequency^19^. However, for the case of the phonon probe, fast transients in amplitude (e.g. due to a material boundary) will be entangled with slow decay mechanisms of the TRBS signal, such as acoustic attenuation in the couplant medium. The slow decay of material attenuation is characterised according to the protocol described in ‘Materials and methods’ and is predicated on the following assumptions: (1) that the water couplant is homogeneous and (2) that the acoustic path length does not extend beyond the focal volume of the probe beam^34^. With the phonon probe, the latter condition is preserved by using a low NA fibre (NA = 0.13); therefore, the optical depth of focus (~50 μm) is much greater than the acoustic path length (~10 μm) and permits an unambiguous measurement of the material attenuation coefficient in the couplant, $\alpha_{{\text H}_2{\text O}}$.

After measuring the attenuation coefficient of the water couplant, CWT converts a TRBS signal into a proximity measurement according to the methodology described in Fig. 2 and ‘Materials and methods’. To briefly summarise the procedure: CWT transforms the TRBS signal (Fig. 2b) into time-varying acoustic amplitude (wavelet coefficients), either at a given frequency (e.g. *f*_B_(H_2_O), blue in Fig. 2d) or a range of frequencies (Fig. 2e). The wavelet coefficients (*C*_w_) are then normalised by the slow decay rate (green in Fig. 2c) provided by the reference signal (Fig. 2a), and a new set of wavelet coefficients (*C*_w_″, Eq. (3)) is obtained (Fig. 2f, dark blue). The normalised *C*_w_″ isolates the edge response and the material boundary is measured at the position in space where the amplitude of *C*_w_″ is half-way between the absolute minimum and maximum of the response (Fig. 2f, vertical line and dots). The strength of contrast in acoustic impedance and photoelasticity between the couplant and object materials will determine the amplitude at the absolute minimum of the *C*_w_″ response (Supplementary Section 1 and Fig. S1b).

A one-dimensional (1D) analytical model, which calculates transient reflectivity due to the presence of strain^39^, was used to corroborate the effectiveness of the proximity measurement technique. The proximity measured in experiment (dark blue dot in Fig. 2f) was used to define the proximity of the fibre-tip and PS interface in the 1D geometry of the model. The simulated signals were processed according to the same proximity measurement protocol as above, resulting in the light blue (and dark red) curves shown in Fig. 2, which closely reproduce the trends observed in experiment. Acoustic impedances of 1.5 and 2.4 kg m^−2^ s^−1^ were used for the simulated water and PS materials, respectively, and the ratio of the photoelastic constants was approximately equal^40,41^.

**Fig. 3:**
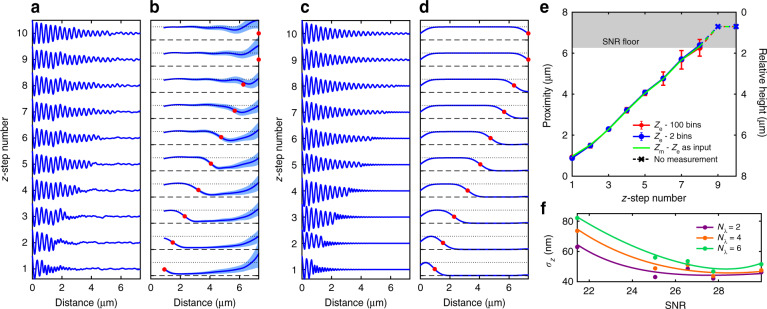
Calibrating the in-depth measurement performance of the phonon probe. **a** TRBS signals as the phonon probe is incrementally stepped away from a PS boundary (vertically offset for clarity). **b** Normalised wavelet coefficient traces at each step, light blue shading represents standard deviation of time-varying amplitude. Red dots mark the proximity measurement (from *z* = 0), and are anchored to the end of the trace when a threshold is not met. Black dashed line is *C*_w_″ = 0 and dotted line is *C*_w_″ = 1. **c**, **d** Simulated TRBS and wavelet coefficient signals using the measurements in **b** as input water layer thicknesses. **e** Comparison of experimental (*z*_e_) and simulated (*z*_m_) proximity measurements showing the increase in precision with signal averaging (e.g. from 100 to 2 bins), yet a decrease in precision with distance. Proximity can be converted to height using a physical floor measurement or relative to the SNR floor. **f** Precision of a proximity measurement (*σ*_*z*_) as a function of SNR and the number of phonon wavelengths contained by the wavelet (*N*_*λ*_)

The ability for the phonon probe to measure changes in proximity was tested by varying the stand-off distance between the fibre-tip and the Petri dish. Figure 3a shows the result of this experiment repeated over 10 *z*-positions (100 measurements at each step). The proximity measurement protocol was applied to the signal from each step using the tenth *z*-position as the attenuation reference (Fig. 3b). The position at which the wavelet amplitude is half-way between the absolute minimum and maximum of the trace was assigned to be the proximity measurement (Fig. 3b, red markers). An additional minimum amplitude threshold (see Supplementary Section 1 for details) was used to distinguish when a measurement could not be made, e.g., when the boundary is beyond the depth-measurement range of the device (Fig. 3b, top two red markers).

Each experimental proximity measurement (*z*_e_) was then used as an input for the distance of water between the transducer and the PS object in simulation, producing the traces in Fig. 3c. Experimental (*z*_e_) and simulated (*z*_m_) proximity measurements from Fig. 3b, d are then collated in Fig. 3e as a function of step number and experimental averaging bin size. Each point on the red curve represents the average measurement and standard deviation of 100 bins each containing one measurement, whereas the blue curve averages the data into two bins each containing 50 measurements. Overall there is strong agreement between experiment and simulation, with the only notable deviation occurring at the first measurement (*z*-step 1), which is likely due to the boundary occurring within the blind zone of the wavelet transform.

Due to the distance-dependence of acoustic attenuation, the signal-to-noise ratio (SNR) of the signal decreases as a function of distance, and therefore the standard deviation of the measurements increases as proximity decreases. The position at which measurements can no longer be distinguished from the end of the time window (dotted portion of Fig. 3e) acts as the SNR floor. Therefore we conclude that the depth-measurement range of the device is estimated by the distance between the closest proximity measurement (in Fig. 3e) *z*_e_ = 0.9 μm and the SNR floor *z*_e_ = 6.4 μm: *z*_DR_ ≈ 5.5 μm. This is ultimately determined by the overall SNR which depends on the specific experimental conditions. Factors such as signal averaging, beam power, and acoustic attenuation influence the SNR, and can be optimised to increase the depth-measurement range. In particular, moving to a longer phonon wavelength (by using a longer probe wavelength) will increase the range significantly since the signal attenuation is proportional to the square of the phonon frequency *f*_B_^2^ (ref. ^42^). For instance, using a probe wavelength of *λ*_probe_ = 1550 nm would improve the nominal depth-measurement range to *z*_DR_ > 20 μm.

The proximity measurement technique presented here can be rendered into a measurement of the relative or absolute height of a microscopic object. When scanning the probe in *x* and *y* (fixed *z*) across the sample plane, if the height of an object is less than the depth-measurement range of the probe, then an absolute height map is created by subtracting the proximity map from its maximum value (the floor). Otherwise, the difference between the proximity map and the SNR floor generates a relative height map (right *y*-axis in Fig. 3e). In the following section this methodology will be applied to the 3D imaging of polymer microstructures.

### Three-dimensional imaging

**Fig. 4:**
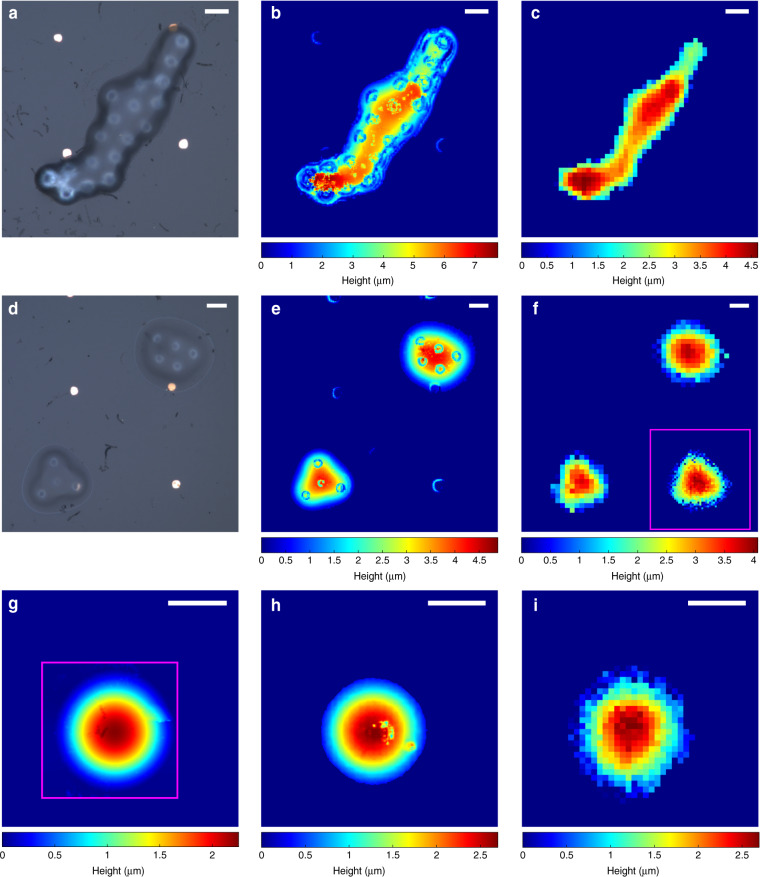
3D picosecond ultrasonic profilometry of microscopic objects. **a**, **d** Optical brightfield images of the scanned PS microstructures. **b**, **e**, **h** Optical profilometry reveals the full height of the microstructures, yet contains artefacts due to the optical inhomogeneity of the objects. **c**, **f**, **i** 3D height reconstruction using the phonon probe for picosecond ultrasonic profilometry. Relative height measurements were achieved for **c**, **f** and a full height reconstruction was possible for **i**. **f** Pink square reveals bottom left object with finer spatial sampling. **g** Profile measured through AFM, with insufficient depth-measurement range to recover the object peak; pink square indicates the AFM field of view. Scale bars are 10 μm

To evaluate the 3D-imaging performance of the phonon probe, 10 μm diameter PS microspheres (Bangs Laboratories, Inc.) were assembled into complex microstructures through drop-casting and partial melting (see Fig. 4a, d). Using the in-depth measurement protocol, the fibre-tip was positioned to within *z* ≈ 2–3 μm of the apparent centre of a microstructure (couplant is a water droplet). This stand-off—which was maintained for the subsequent area scan—is a compromise between maximising depth-measurement range (when stand-off is minimised) and leaving enough space to accommodate for height fluctuations of the object.

An area of 100 μm × 100 μm was scanned in 2 μm steps over the course of 2.8 h (<4 s per pixel, 15,000 oscilloscope averages), during which time the water droplet couplant did not fully evaporate. The proximity measurement protocol was repeated for each pixel generating a proximity map between the fibre-tip and the object, which was then converted to height (relative or absolute) by subtracting the proximity at each pixel from the least proximal value in the map (image processing detailed in Supplementary Section 3 and Fig. S3). Figure 4c presents a 3D image of a PS microstructure (shown in Fig. 4a) acquired using the phonon probe for PUP. Since the true height of the object is taller than the depth-measurement range of the probe, the map shows the profile of the upper *h*_pup_ = 4.63 μm of the object. An optical profiler was used to verify the true height of the object, *h*_abs_ = 7.75 μm, as shown in Fig. 4b. Despite the mismatch in depth-measurement range, the phonon probe effectively reproduces the general profiles measured through optical profilometry.

**Fig. 5:**
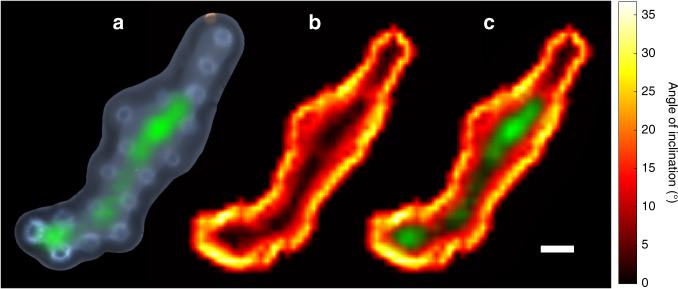
Spatially resolved Brillouin spectroscopy with a fibre probe. The mean Brillouin frequency for the structure in Fig. 4a was measured as *f*_B_(PS) = 8.43 ± 0.17 GHz; **a** spectral amplitude map at *f*_B_(PS) overlaid onto the brightfield image. **b** Surface gradient (degrees inclination) revealing the steep edges of the object. Given the refractive index contrast (optical and acoustic) between water and PS, *f*_B_(PS) measurements are possible when the surface gradient of the object varies by less than 10°. **c** This is confirmed by overlaying the amplitude map (**a**) onto the surface gradient map (**b**), where high amplitude corresponds with flatness. The object Brillouin frequency is unresolved for the shortest region of the phantom due to attenuation. Scale bar: 10 μm

In addition to topographical information, measurements of the object Brillouin frequency were also attainable as the ultrasound coupled into the PS structure. An average frequency of *f*_B_(PS) = 8.43 ± 0.17 GHz was measured in the microstructure, and the spatial variation in amplitude for this frequency range is mapped in Fig. 5a. When the optical and acoustic beams are incident on the PS structure, if the beams refract away from each other inside the PS, the volume and strength of the TRBS interaction will be reduced. Therefore, the ability to measure sub-surface Brillouin oscillations strongly depends on the velocity contrasts (optical and acoustic) between the two materials, and the surface gradient of the object (Fig. 5b). We estimate from experiment that the maximum angle of inclination for measuring *f*_B_ in a PS object is ≈10°. Measuring sub-surface elasticity will also depend on the amount of attenuation experienced before sound reaches the object; this is relevant for shorter regions of the object, e.g., the top right feature in Fig. 4c is sufficiently flat (top right in Fig. 5b), yet *f*_B_(PS) is unresolved (top right in Fig. 5c). For the case of biological materials (reduced velocity contrasts with water) the differences in refraction angles will be reduced, and we anticipate there will be a much greater range of acceptable surface gradients. Refer to Supplementary Section 2 for a deeper discussion on this topic. Overall, this demonstrates the parallelised spectroscopic capability of the probe, which can be utilised in a similar manner as conventional Brillouin spectral imaging^14^.

A full-height reconstruction of an object is possible when its peak height is shorter than the depth-measurement range of the probe. Figure 4f shows the output of the phonon probe when this condition is approached. The maximum height measured acoustically, *h*_pup_ = 3.85 μm, is nearly a micron shorter than that measured with optical profiling (Fig. 4e), *h*_abs_ = 4.89 μm, indicating that a relative height measurement was made. However, the mismatch in lateral resolution between the two techniques is likely contributing to this offset. When the tallest features of the object vary with spatial periods shorter than the lateral resolution of the phonon probe, the true height of the feature will be effectively aliased. Additionally, the lower-left structure (Fig. 4d) was re-scanned with higher resolution *x/y* scanning (Δ_*xy*_ = 1 μm), resulting in the pink box in Fig. 4f. Despite an apparent increase in image resolution, this is not the case since the optical point spread function has not been altered; instead, it can be said that the precision of the pixel values has increased. The probe beam will dwell over any given μm^2^ area for approximately four times longer than previously, quadrupling the number of averages acquired within this patch.

Figure 4i presents the case in which the depth-measurement range of the phonon probe exceeds the height of an object. The maximum height measured in this instance was *h*_pup_ = 2.695 μm, which demonstrates good agreement with the optical profilometry measurement of *h*_abs_ = 2.700 μm. Atomic force microscopy (AFM) was also performed on this particular object, as shown in Fig. 4g. Despite the AFM having insufficient depth-measurement range to corroborate the measurements from the optical profiler and phonon probe, the *h*_AFM_ = 2.25 μm of available height still corroborates the surface variation and geometry detected by the fibre device.

### Performance characterisation

The lateral resolution of the device is determined by the mode field radius of the single-mode optical fibre, *r*_PSF_ ≈ 2.5 μm. Due to the low NA of the fibre, NA = 0.13, the decrease in resolution from defocus is negligible Δ*r*_PSF_ < 100 nm for the distances covered by this study. When used for 3D-sectioning purposes (e.g. using *f*_B_ as a contrast mechanism), the probe is capable of super-optical axial resolution since the sampled phonon wavelength is sub-optical by a factor 1/2*n*_medium_. In this context, axial resolution will depend on the axial distance spanned by the wavelet (e.g. the width of the edge response in Fig. 2f) which acts as the impulse response of the system. Using a wavelet width of *N*_*λ*_ = 6 phonon wavelengths (~1.8 μm), the configuration presented in this work is capable of *z*_min_ = 960 nm axial resolution, based on the metric in ref. ^28^. Longer wavelets will aid in resolving subtle shifts in Brillouin frequency at the cost of reduced axial resolution. However, similar wavelet parameters as above have proven capable of resolving sub-cellular axial features in adipose and 3T3 fibroblast cells^28,43^.

Due to the reliance on measuring sound velocity, the precision with which the Brillouin frequency can be measured ultimately affects the ability to localise the object-boundary in space, or measure changes in elasticity. To this end, we have demonstrated previously that the phonon probe is capable of 4 MHz frequency precision, and Δ*f*_B_ = 10 MHz frequency resolution which translated to a Δ*v* ≈ 5 m/s shift in sound velocity (and acoustic impedance)^33^. These quantities are dependent on the SNR of the signal, and therefore can be improved with signal averaging or transducer efficiency. Such resolution becomes crucial when there exists low-frequency contrast between couplant and object materials. In particular, biological targets such as eukaryotic cells have been shown to exhibit frequency contrasts (depending on cell type and probing wavelength) of approximately Δ*f*_B_ = 0.3–1 GHz with the surrounding medium, and Δ*f*_B_ = 50–200 MHz between major cellular components such as cytoplasm, nucleus, and nucleolus^15,18,28^. Additionally, objects with large frequency contrast, e.g., *f*_B_ > 70 GHz for high-pressure ice (*v* = 15,500 m/s)^44^, are still within reach of the CAP bandwidths probed through TRBS.

Given the profiling capability shown in this work, a different criterion than axial resolution applies: the surface in any given voxel can be treated as a single object, and therefore the concept of boundary localisation precision (*σ*_*z*_) is more pertinent. Figure 3f shows the standard deviation of 20 proximity measurements as a function of the SNR of the TRBS signal (greater SNR achieved through signal averaging) and the number of phonon wavelengths (*N*_*λ*_) spanned by the wavelet used for time-frequency analysis. As more cycles of the wavelet are included in the transformation, the precision with which the instantaneous frequency is measured increases. Given the trade-off between temporal and frequency resolution in CWT, the increased frequency precision comes at the cost of decreased temporal (and spatial) precision. In this work the phonon probe was capable of measuring proximity with a minimum precision of *σ*_*z*_ = 45 nm (Fig. 3f), which is over an order of magnitude smaller than the optical wavelength and ~3 orders of magnitude smaller than the depth of focus of the optical fibre. As the acoustic impedance or Brillouin frequency contrast between the couplant and object begins to decrease (e.g. for a biological material), it is likely that this profilometric precision decreases as the amplitude roll-off of *C*_w_″ begins to approach the amplitude of the noise floor. However this could also be remedied by increasing SNR, e.g., at the cost of acquisition speed.

A key source of systematic error arises from the temperature fluctuations surrounding the photothermally excited transducer tip^33^. When the measurement volume contains a temperature gradient, the refractive index and sound velocity of the couplant medium (water) will vary spatially due to thermo-optic and thermo-acoustic effects. In the TRBS signal, these effects manifest as changes in the instantaneous Brillouin frequency (in the time domain), which then quantifies the variation in instantaneous sound velocity (approximately). The time-varying sound velocity can then be used to create a 1D spatial vector (for depth measurements) which is discretised inhomogeneously, i.e., at the *i*th time sample Δ*z*_*i*_ ≠ Δ*z*_*c*_ = *v*_*c*_Δ*t*_*i*_ where the subscript *c* implies constant. Consequently, a measurement made using a homogeneous spatial vector (*z*_*c*_) will differ from that using the inhomogeneous spatial vector (*z*_*T*_), producing a systematic error *ε*_*z*_(*t*) = *z*_*c*_*(t)* – *z*_*T*_(*t*) as a function of signal time. The proximity measurements presented here were performed using a homogeneous spatial vector with spacing determined by the weighted average sound velocity in the couplant medium; this was to avoid discontinuities that occur when measuring the instantaneous sound velocity (see Supplementary Fig. S4b). The calibration of this systematic frequency error (presented in Supplementary Section 4) allows us to re-sample the spatial vector, Δ*z*_*T*_ = Δ*z*_*i*_(*f*_B_) = *v*_*i*_(*f*_B_)Δ*t*_*i*_, and provide an estimate of *ε*_*z*_. As it pertains to the height maps in Fig. 4, the following maximum errors were calculated: |*ε*_*z*_| = 90, 70 and 40 nm (respectively from top to bottom).

### Phonon imaging bundle

One of the most promising aspects of the phonon probe is that the single-fibre design, transducer fabrication, and scanning mechanism can be extrapolated to *N*-number fibres, e.g., a coherent imaging bundle. Employing an imaging bundle for this purpose would partially simplify the *x/y* scanning requirements of the probe since the distal end could be left stationary and the pump and probe beams scanned (one core at a time or multiple) across the proximal end. Consequently, wide fields of view (e.g. ~1 mm) could be routinely achieved without needing to move the device.

**Fig. 6:**
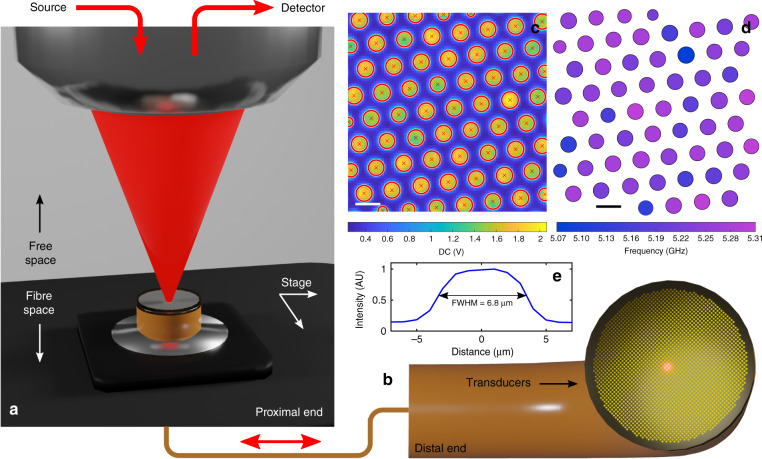
Time resolved Brillouin scattering with a standard optical fibre imaging bundle. **a**, **b** Proximal and distal ends of the phonon imaging bundle; distal facet contains 18,000 transducers. Each core serves as a pixel which is read-out sequentially through proximal scanning. **c** Optical intensity (DC) map for centroiding the cores. Calibration of core positions in **c** allows quick scanning and measurement of TRBS and Brillouin frequency (in water) at the distal end (**d**). **e** Optical intensity distribution along the cross-section of a single core in **c**. Scale bars are 10 μm

An experimental description of the 18,000 pixel phonon imaging bundle prototype (Fig. 6a, b) can be found in ‘Materials and methods.’ Initially, an 80 μm × 80 μm area at the proximal end was scanned by the probe beam, capturing the intensity map in Fig. 6c. Consequently, a full-width half-maximum value of FWHM = 6.8 μm was determined for the optical response of a core (Fig. 6e); however, the 11.3 μm spacing between cores is the limiting factor for lateral resolution. The centroids of the cores were determined and used to set the coordinates for the TRBS scan (*λ*_pump_ = *λ*_probe_ = 780 nm). The distal end of the bundle—containing 18,000 transducers—was placed in water and the same field of view was scanned in 4 min resulting in the Brillouin frequency map found in Fig. 6d. Despite coupling into the bundle channels with a lens containing similar numerical aperture as the imaging bundle (see ‘Materials and methods’), and thus exciting unwanted higher order modes and attenuation, the bundle still proved effective for the TRBS process in water.

## Discussion

To the best of our knowledge, the phonon probe is the highest resolution ultrasonic 3D fibre-imaging device in the world^45^, with a lateral resolution *r*_PSF_ ≈ 2.5 μm set by optics and the following performance metrics provided by acoustics: axial precision of *σ*_*z*_ = 45 nm, and a theoretical axial resolution of *z*_min_ = 960 nm. Moreover, axial precision and resolution are quantities that can be further improved as SNR is optimised. Even beyond acoustics, this device is uniquely positioned within the landscape of optical fibre-imaging systems. The sub-micron axial resolution is over an order of magnitude higher resolution than many leading endomicroscopy techniques^3,46^, and the phonon probe achieves this without confocal scanning and, importantly, without fluorescent labels. This level of performance has allowed the phonon probe to attain 3D information on length-scales best accessed by state-of-the-art bench-top profilometry—atomic force microscopy and optical profiling—which has led to a novel profiling technique: PUP. Compared with high-power objective lenses used for optical profilometry (370 nm lateral resolution in this work), or nanometre-sized AFM tips, the phonon probe is limited in its ability to laterally resolve sub-micron features. However, numerous routes exist for further increasing resolution with high-frequency ultrasound: tapering the fibre-tip, designing acoustic lenses^47,48^ or even structuring the phonon field with nanoscopic mechanical resonators^49^.

We believe that the phonon probe offers unique features—such as non-contact operation and endoscopic potential—that are supplementary to AFM and optical profiling. Two further examples are evident in this study. (1) The optical inhomogeneities in the polymer microstructures (Fig. 4a, d) introduce inhomogeneous phase shifts to the reflected light used for interferometry in optical profiling^50,51^, and ultimately results in measurement artefacts (visible in Fig. 4b, e). In practice, this is typically mitigated by coating a highly reflective opaque material onto the sample, such as gold, which is non-biocompatible^51^. With the phonon probe, sub-surface features remain time resolved from the surface (when acoustic impedance mismatch is low between background medium and object), or are undetectable when acoustic impedance mismatch is high or the optical and acoustic fields lose spatial overlap (Supplementary Section 2). (2) The atomic force microscopy performed in this paper was limited by both the travel range of the piezo-element of the instrument and the cantilever/tip dimensions; in this instance the depth-measurement range was less than 2 μm (Fig. 4g); however, it is common for AFM instruments to achieve greater depth than this. This points towards a general need in AFM measurements for re-tooling or utilising a completely separate instrument, and is especially relevant for application in biological cell imaging^52^. Although the *z*_DR_ = 5.5 μm depth-measurement range of the current phonon probe is only a factor 2 longer than the AFM used, this can be extended in the following ways: using longer probe wavelengths (*z*_DR_ ~ *λ*_probe_^2^), increasing SNR (through transducer efficiency, beam power, or signal averaging), or utilising the *z*-position of the fibre-tip as a degree of freedom in 3D imaging.

High-resolution performance for our single-fibre device is enabled by distally scanning the sample with micron-resolution in *x/y* with respect to the fixed probe. For the configuration reported in this work, *z*-motion is only required to initially localise the imaging target since the acoustic time-of-flight is alone sufficient for in-depth reconstruction. In addition, we foresee no major complications with assigning the *x/y* scanning behaviour to the fibre-assembly in a bench-top configuration of the phonon probe. The physical constraints imposed by the scanning equipment impact the overall footprint of the optical fibre transducer which itself is 125 μm in diameter. However, the development of miniaturised distal scanning equipment is commercially relevant and therefore an area of active research which is poised to enable in vivo articulation^1^. We re-emphasise that a key benefit of the phonon probe technology is its potential for extrapolation to imaging bundles, which relaxes the requirement for high-resolution distal scanning in future in vivo environments.

In contrast with similar transducer-based techniques that generate and detect coherent phonon fields^17–19,29^, the device presented here demonstrates that direct contact between the transducer substrate and specimen is no longer compulsory, and is therefore the first truly non-contact device of its kind. However, as with endoscopic ultrasonic techniques in general, non-contact operation is predicated on the use of a couplant medium (naturally occurring or otherwise), especially in a biomedical context. The novelty of the device can also be extended to the greater field of Brillouin spectroscopy and microscopy; for the first time we have demonstrated that a GHz frequency fibre probe is capable of resolving multiple elastic signatures within a single pixel, and therefore within a 3D environment (Fig. 5a).

Looking forward from this perspective, the phonon probe is promisingly situated for application to non-contact and label-free 3D elastography—of mammalian cells containing lateral dimensions of 10–100 μm—with sub-cellular depth resolution. For such imaging targets, tolerance to steep topographic gradients (Fig. 5b) will be increased due to the low refractive index contrast (optically and acoustically) between biological cells and aqueous media (see Supplementary Fig. S2), and will enable mapping the cell Brillouin frequency^18^ (additional colours would arise in Fig. 5a). However, any application to biological samples will require careful consideration of the transducer heating^33^. Improving the SNR of the technique will enable cooler operation, as will increasing the depth-measurement range and thus allowing a greater stand-off between heat-source and specimen.

Within industrial and aerospace manufacturing there is great demand for surface inspection probes for components with complicated geometries and very little optical contrast; this would be a natural pairing, in part due to the automated and roboticised nature of current inspection techniques. In a biological capacity the phonon probe is well suited for ex vivo inspection and imaging applications where a bulky microscope body is inconvenient, e.g., when other instruments, such as an extruder or print-head, obstruct the optical axis. Burgeoning technologies such as 3D bio-printing and tissue engineering^53^ could utilise the phonon probe as an inline inspection tool by integrating it directly to the outer diameter of the print-needle. Furthermore, this could be fulfilled with high resolution and label-free contrast, qualities which offer value throughout the life sciences. The future in vivo potential for our device could be realised by using the phonon imaging bundle as a contact probe for cellular resolution tissue diagnostics, and in settings where commercially available contact endocytoscopes are used^1,2,6^.

## Materials and methods

### Phonon probe set-up

The phonon probe system uses asynchronous optical sampling^54^ to synchronise 100 fs pump and probe pulses (Spectra-Physics, Tsunami) for TRBS excitation and detection. Once light is coupled from free-space to fibre (Thorlabs, FiberPort), a series of single-mode patch cables (Thorlabs, 780HP) relay through a 2:2 coupler (Thorlabs) and wavelength division multiplexer (OZ Optics) to combine the pump (*λ*_pump_ = 730 nm) and probe (*λ*_probe_ = 850 nm) pulses into a common channel (see Fig. 1a). The final common channel consists of a single-mode custom patch cable (Thorlabs, 780HP) with an FC/PC proximal termination, bare-fibre distal termination, 5 μm diameter core, 125 μm diameter cladding, NA = 0.13, and 780 nm centre-wavelength. A 15 nm gold transducer is sputter coated onto the flat distal end in order to optically absorb pump pulses and thermo-elastically generate ultrasound.

The raw TRBS signal undergoes initial signal processing based on the protocol described in ref. ^55^. This process is briefly summarised as: (1) removal of pump-probe coincidence peak which is set as time zero, (2) polynomial fitting and subtraction of low-frequency thermal response, and (3) digital low-pass filtering. The resulting modulation in reflectivity (Δ*R*, Fig. 1c) can then be analysed in the frequency domain by fast Fourier transform (Fig. 1d).

In order to access spatio-elastic information with high resolution, the phonon probe system was outfitted with the following distal-scanning equipment: linear stages to control the *x/y* position of the specimen stage (PI, M-605), and a piezo nanopositioner (PI, P-721 PIFOC) to manipulate the axial position of the fibre-tip. Additionally an inverted brightfield microscope was arranged beneath the sample to provide an optical aid for localising the fibre-tip with respect to the sample plane.

### Wavelet transformation and normalisation

Before wavelet transforming the signal of interest, its frequency content is evaluated by fast Fourier transform; Brillouin frequency peaks (e.g. *f*_B_(H_2_O)) are then used to determine the scaling factors of the mother wavelet, which was chosen to be a complex Morlet:

2 $$\Psi \left( t \right) = \frac{1}{{\sqrt {\pi f_{\mathrm b}} }}{\mathrm e}^{2i\pi f_{\mathrm c}t}e^{\frac{{t^2}}{{f_{\mathrm b}}}}$$

where *f*_b_ and *f*_c_ are parameters for the bandwidth and centre frequency of the wavelet, respectively. The bandwidth of the resulting daughter wavelets (set by *N*_*λ*_) in part determines the SNR of the transformation as well as the axial resolution.

The magnitude of the wavelet transformation of a TRBS signal at *f*_c_ = *f*_B_(H_2_O) results in a set of wavelet coefficients (*C*_w_), which can be characterised as having the following responses (refer to Fig. 2c): (1) an initial rise in amplitude (blind zone) which occurs as the initially clipped wavelet begins to sample the TRBS signal, followed by (2) a slow amplitude decay owing primarily to the acoustic attenuation rate in water, $\alpha_{{\text H}_2{\text O}}$ = 0.30 ± 0.06 μm^−1^, which was measured by fitting to an exponential function (Fig. 2c, green curve) and agrees with literature^42^. (3) If a boundary is present (Fig. 2d) the *f*_B_(H_2_O) mode experiences an abrupt decay, which is accompanied by an increase in amplitude of the *f*_B_(PS) mode (red curve) which then attenuates at a rate of *α*_PS_ = 0.83 ± 0.07 μm^−1^.

In order to extract a spatial measurement from the transition region, it is convenient to normalise both the high and low-frequency amplitudes by the experimental attenuation rate of each material:

3 $$C_{\mathrm{w}}^{{\prime\prime}}\left( t \right) = C_{\mathrm{w}}\left( t \right)e^{\alpha vt}$$

where *α* and *v* are material specific. It then follows that the distance at which the low-frequency (*f*_B_(H_2_O)) amplitude falls to 50% (between the absolute maximum and minimum amplitude values) correlates with the wavelet being centred on the material boundary, and can be used as a measurement of proximity with respect to the fibre-tip. In principle, this same criterion can be applied to the high-frequency boundary transition (red dots in Fig. 2f); however, in practice this is more difficult as the amplitude in the second material is dependent on a number of competing effects: (1) the acoustic impedance mismatch of the two materials, (2) the photoelastic constant, (3) material attenuation which preceded the boundary, and (4) the topography of the object, all of which adversely affect SNR. For these reasons, this study was carried out utilising solely the roll-off position of Brillouin oscillations in the carrier medium.

### Fibre bundle experiment

The imaging bundle used in this study was a Schott leached fibre bundle with the following specifications: 1.65 mm outer diameter, 1.35 m total length, 11.6 μm diameter cores, 18,000 total pixels, and per-pixel NA = 0.39. Based on these parameters it is estimated that any given core can support *M* ≈ *V*^2^/2 ≈ 170 modes (at *λ*_probe_ = 780 nm), where *V* is the normalised frequency parameter. Similar to the single-fibre probe, 15–20 nm of gold was deposited onto the distal end of the imaging bundle. Light was coupled from free-space to an individual core by fixing the proximal end of the bundle within the focal plane of a microscope objective lens (NA = 0.45). This process is varied in space by translating the proximal end with high-resolution *x/y* stages (Thorlabs, MLS203-1), but can also be achieved by fixing the position of the proximal end and raster scanning the beams. For detection, a 50/50 beam-splitter and detector were placed in the pump-probe input path in order to tap-out the reflected probe signal after it has Brillouin scattered beyond the distal end and re-coupled into the bundle.

## Supplementary information

Supplementary information for: Phonon imaging in 3D with a fibre probe
