# Supplementary material for: Phonon imaging in 3D with a fibre probe
Source: Light Sci Appl. 2021 Apr 27;10:91. doi: 10.1038/s41377-021-00532-7 (PMC8079419; doi:10.1038/s41377-021-00532-7)
Supplement: Supplementary file 1 — Supplementary information for: Phonon imaging in 3D with a fibre probe [file 41377_2021_532_MOESM1_ESM.pdf]

**Supplementary information for:**

# Phonon imaging in 3D with a fibre probe

**Salvatore La Cavera III*, Fernando Pérez-Cota, Richard J. Smith, and Matt Clark**

Optics and Photonics Group, Faculty of Engineering, University of Nottingham, University Park, Nottingham, NG7 2RD, UK

*Corresponding author: salvatore.lacaveraiii@nottingham.ac.uk

## 1. Proximity measurement criteria

After the time resolved Brillouin scattering (TRBS) signal has been wavelet transformed ($C_w$), $C_w$ is normalised ($C_w$") in order to remove the effect of material attenuation (see manuscript Fig. 2f and Eq. 3). Following normalisation, when the wavelet (at frequency $f_B(H_2O)$) has fully left the water medium, $C_w$" should transition from 1 to a minimum amplitude ($m_C$) within the distance spanned by the wavelet. The centroid of this edge response is then taken to be the distance measurement between the fibre-tip and the object-boundary. The value of the minimum amplitude ($C_w" = m_C$) will depend on the following mismatches between the two materials: acoustic impedance, photoelastic coefficient, and Brillouin frequency (frequency sampled by a single wavelet). For example, the acoustic impedance mismatch between water and a PS object will result in a partial reflection of the $f_B(H_2O)$ mode, and TRBS will be detected for the counter-propagating mode, as seen in the non-zero amplitude in the $f_B(H_2O) \sim$ 5 GHz band in manuscript Fig. 2e (from $z \approx 3 \rightarrow 5$ μm). However, considering the ideal case in which $m_C \rightarrow 0$, when a boundary transition between water and the object has occurred, the proximity measurement should be recorded at the half-way point (amplitude $\tau_{half}$) between amplitudes 0 and 1, $\tau_{half} = 0.5$ (shown in Fig. S1a). For the case when $m_C > 0$ (e.g. with weakly contrasting objects), $m_C$ is first determined (green circle in Fig. S1b), and the amplitude half-way between this point and $C_w" =$ 1 serves as the proximity measurement ($\tau_{half} > 0.5$): $\tau_{half} = (1+m_C)/2$. Therefore it is appropriate for the user to define an amplitude threshold $\tau_{min}$. If $m_C < \tau_{min}$ a measurement will occur (Fig. S1b); otherwise, register the pixel as a non-measurement by assigning the proximity measurement to be the

end of the TRBS signal time-vector (Fig. S1c). This criteria was used to distinguish the immeasurable pixels in manuscript Figs. 3b and 3d ($z$-step numbers 9 and 10).

The SNR floor of the phonon probe, and therefore its measurement range, is partially set by the least proximal distance measurement that meets the amplitude threshold. In manuscript Figs. 3b and 3d, for both experiment and simulation this criterion was finally broken after $z$-step number 8, resulting in an SNR floor of $z \approx 6.4$ µm. Since the most proximal measurement registered was $z \approx 0.9$ µm (at step 1), an effective measurement range of $z_{\mathrm{DR}} \sim 5.5$ µm was assigned to this configuration of the phonon probe.

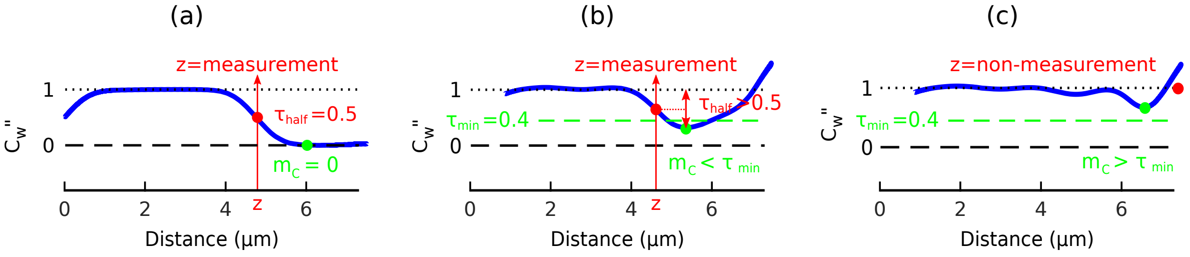


**Figure S1.** Threshold criteria for making a proximity measurement. (a) Ideal scenario: the absolute minimum ($m_C$, green circle) for the normalised wavelet coefficients falls to amplitude zero, in which case a proximity measurement is recorded at the position of amplitude $\tau_{\mathrm{half}} = 0.5$ (red circle). (b) In reality $m_C$ will not fall to zero, e.g. due to acoustic impedance contrast, yet a measurement can still be attained; an amplitude threshold $\tau_{\mathrm{min}}$ is then defined, if $m_C < \tau_{\mathrm{min}}$ a measurement is recorded at $\tau_{\mathrm{half}} = (1{+}m_C)/2$. (c) If the threshold is not met the signal will be labelled as having not recorded a measurement, and $z$ anchored to the end of the time-vector.

## 2. Measuring object Brillouin frequency

When the object under investigation has a curved topography with steep inclinations or considerable surface roughness (Fig. S2a), both the optical probe beam and the acoustic beam will refract at oblique interfaces. If there exists a contrasting velocity gradient experienced by the photons and phonons (Fig. S2b), optical and acoustic rays will refract in opposite directions (towards and away from the normal to the surface), thus causing the optical and acoustic fields to diverge from each other. This will additionally lead to a shift towards lower Brillouin frequencies due to the angle ($\Delta\theta$) between the optical and acoustic wavevectors:

$$f_{\mathrm{B}} = \frac{2nv}{\lambda_{\mathrm{probe}}} \cos(\Delta\theta) \tag{S.1}$$

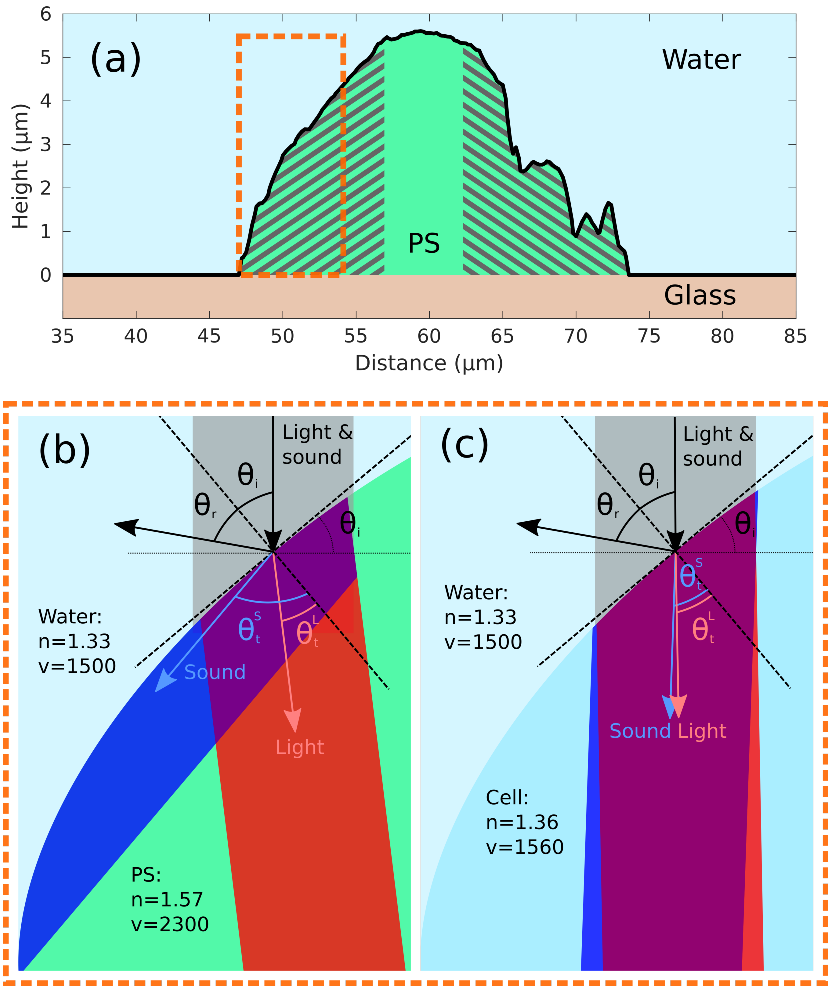


**Figure S2.** Divergence between optical and acoustic fields at oblique scattering interfaces. (a) Cross-sectional profile of the PS microstructure in manuscript Fig. 4b (from optical profilometry). The shaded regions indicate where the inclination angle is too steep to experimentally measure $f_B$(PS). Orange box outlines a region in which the surface normal to the object creates an angle of $\theta_i \approx 40^\circ$ (b) with respect to the normally incident acoustic and optical wavefronts (black arrow from top). Snell's law was used to calculate the angles of refraction ($\theta_t$) for the acoustic (blue) and optical (red) rays (approximate spot sizes shown by blue and red regions surrounding the ray). The region of opto-acoustic interaction (purple) is reduced for the case of a PS object causing a reduction in TRBS signal amplitude. (c) For a biological cell, $\theta_t$ will more mildly deviate from the angle of incidence (e.g. for the acoustic ray) and therefore the opto-acoustic interaction will not be affected as significantly.

The degree to which the acoustic (blue in Fig. S2b) and optical (red in Fig. S2b) fields overlap (purple in Fig. S2b), along with the amplitude and intensity (respectively) of the fields, in part determines the amplitude of the TRBS signal[1,2]. If the volume of this overlapping region decreases with axial distance, the TRBS signal amplitude will experience premature decay as a function of signal-time. The following additional mechanisms for signal loss can also be attributed to large differences between optical/acoustic wavevectors: Brillouin scattered probe light missing the acceptance cone of the optical fibre, probe light being within the acceptance cone but missing the core, or total internal reflection at the PS/water interface. Considering these effects, one would expect that when scanning across the surface of an environment, that elastic information (measuring object Brillouin frequency) can only be attained when the surface is sufficiently flat, or impedance mismatch is sufficiently low, thus ensuring similar optical/acoustic wavevectors. Using the height gradient of a PS phantom (manuscript Fig. 5b) and the location of the measurable Brillouin frequencies (amplitude map in manuscript Fig. 5a), it is estimated that the maximum angle of inclination at which $f_\mathrm{B}$(PS) can be measured is $\theta_\mathrm{i} \approx 10^\circ$ (non-shaded region in Fig. S2a). Beyond this angle in experiment, the spectral peaks for the PS-object Brillouin frequency (shifted by $\Delta f_\mathrm{B} \sim 100$ MHz for $\theta_\mathrm{i} \approx 10^\circ$) were unresolvable from the noise floor. Surface roughness of the object in comparison to a large optical probe spot, will also reduce TRBS signal amplitude since the wavefronts will refract in the form of speckle patterns, therefore reducing the opto-acoustic interaction.

We will now consider how strongly the decrease in opto-acoustic overlap will manifest when the acoustic and optical velocity mismatches are much weaker (than is the case between water and PS), e.g. between water and a biological cell. For example, consider a water medium with the following material properties: $n_{\mathrm{H_2O}} = 1.33$, $v_{\mathrm{H_2O}} = 1500$ m s$^{-1}$, and $\rho_{\mathrm{H_2O}} = 998$ kg m$^{-3}$ (refractive index, sound velocity, and mass density). Now consider a model biological cell constructed using material properties from 3T3 fibroblast cells and white blood cells: $n_\mathrm{cell} = 1.36$ (ref.[3]), $v_\mathrm{cell} = 1560$ m s$^{-1}$ (ref.[4]), and $\rho_\mathrm{cell} = 1085$ kg m$^{-3}$ (ref.[5]). Next, the contrasts in optical refractive index and acoustic impedance ($Z = \rho v$) between water and the model-cell are estimated: 2% and 10% variation (respectively) relative to the values for water. These values are significantly lower compared with the equivalent calculations between water and PS: 20% and 50% variation. Using Snell's law (for optical and acoustic rays), and the above material properties, we estimate that a cell wall would need to contain $\theta_\mathrm{i} > 60^\circ$ inclination to achieve the same loss of overlap (difference in refraction angles) produced by a $\theta_\mathrm{i} = 10^\circ$ inclination of PS. Therefore, we expect that the phonon probe will be able to probe much deeper into weak contrast

objects (cells), and over a wider range of surface gradients, since the optical and acoustic beams remain coincident for longer distances than high contrast objects.

## 3. Image processing for proximity and height maps

The criteria in Supplementary Section 1 were applied to the wavelet transformation of the TRBS signal at each pixel in the area scan. Measurements which did not meet the threshold were classified as “non-measurements" (dark red in Fig. S3a). A binary mask was created by thresholding the proximity map (Fig. S3b). The TRBS signals at the pixels rejected by the mask were collectively averaged into a super-pixel (Fig. S3c) with which the SNR of the object-floor can be increased. If the proximity measurement of the super-pixel does not meet the aforementioned amplitude threshold then the entire image is classified as a relative proximity map. Otherwise, the proximity measurement of the super-pixel (red marker in Fig. S3c) is assigned as the “floor" measurement (dark red in Fig. S3d). Proximity is converted to height by subtracting the proximity at each pixel from the least proximal value in the map (see Fig. S3e).

Classification of relative height or absolute height is similar to atomic force microscopy. With the phonon probe, the user will set an amplitude threshold as discussed in Fig. S1. The user will be alerted when this threshold is not met, thus implying relativity, in a similar way that an AFM user will be alerted that the position of the cantilever/tip is at the limit of the piezo-stage (relative height measurement).

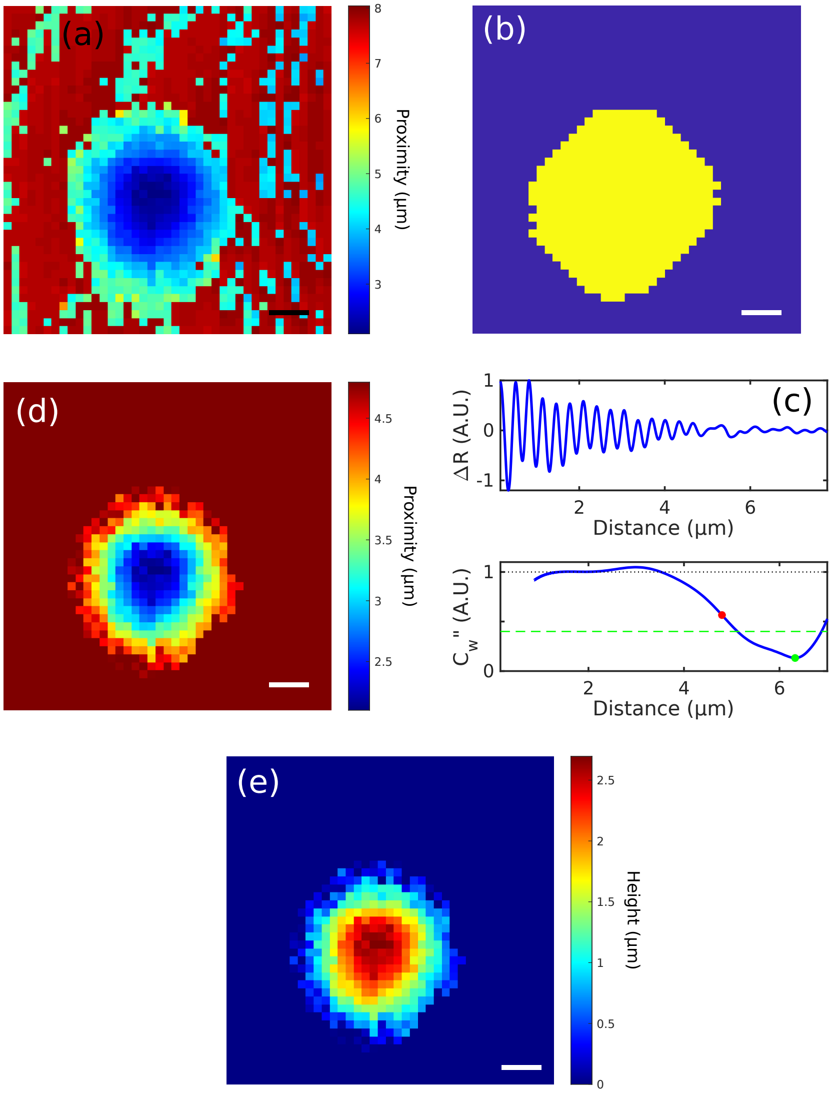


**Figure S3.** Imaging processing for the phonon probe. (a) A set of measurements (non-red pixels) and non-measurements (red pixels) is rendered into a proximity map. A proximity threshold is applied to (a) in order to create a binary mask (b). TRBS signals occupying blue pixels of the map in (b), are averaged (top (c)); a proximity measurement is then performed on the averaged signal in order to measure the distance from the fibre-tip to the substrate (bottom (c)) which is then applied as the "floor" (dark red) of the final proximity map in (d). (e) Each proximity measurement in (d) is subtracted from the value of the floor to create a height map. Scale bars 5 µm.

## 4. Frequency error calculation

In the manuscript, the temporal position of a boundary transition (in the normalised wavelet coefficients of a TRBS signal, $C_w''$) is converted into a spatial measurement by multiplying the average sound velocity measured in the couplant medium (water). This was accomplished by taking the FFT of the entire TRBS signal, and recording the frequency with maximum amplitude within the expected frequency band ($f_B \sim 4.8$ GHz for water and $\lambda_{probe} = 850$ nm); then this frequency measurement is applied to Eq. S.1 in order to solve for $v$, given that $\Delta\theta = 0$ and $n \approx 1.33$ in water.

In reality the Brillouin frequency itself varies as a function of signal-time; one likely cause of this, is that the photothermally excited transducer non-uniformly heats the surrounding water medium. When heated, the refractive index and sound velocity of water fluctuate due to thermo-optic and thermo-acoustic effects. Through Eq. S.1 these effects will modulate the Brillouin frequency measured along the time-of-flight, and therefore the measured sound velocity. For the case of the phonon probe, the temperature rise generated by the transducer diffuses exponentially moving away from the tip[6]. If the sound velocity is varying as a function of axial distance, then so too is the discretisation of the spatial-vector. The result of this frequency or velocity error is a systematic error on the spatial position of the boundary measurement.

To estimate the magnitude of this error, continuous wavelet transformation (CWT) is used to quantify the maximum amplitude frequency along the TRBS signal time-of-flight (Figs. S4a-b). The mother wavelet used was a complex Morlet with a width of $N_\lambda = 6$ complete cycles of the acoustic wavelength; these are the same parameters utilised in the manuscript to allow investigation of the error associated with this wavelet. The transformation process was performed for three separate TRBS signals: two signals which did not contain object boundaries within the measurement volume (NB, blue and red curves in Fig. S4a), and one signal which did contain an object boundary (WB, green curve in Fig. S4a). Within the first hundreds of picoseconds of the signals, the SNR of the frequency measurement is low since the wavelet has not fully entered the time window. All three signals demonstrate a flat frequency response centred at $f_B \approx 4.8$ GHz within the first 1.5 ns of the time window. This well behaved region will contain the highest amplitude portion of the TRBS signal (since the acoustic mode is minimally attenuated) and contributes most significantly to the $f_B$ measurement of the signal as a whole. When $t > 1.5$ ns for the WB-case, the wavelet with centre frequency $f_c = f_B$ no longer contains the maximum amplitude since the acoustic field has been scattered beyond the measurement volume by the object (dotted portion of the curve). Interestingly the frequencies of maximum amplitude begin

to fluctuate beyond $t > 2$ ns for the red and blue signals. If cooling was the only event taking place, one would expect the instantaneous Brillouin frequency to increase with time considering the initial temperature rise of the transducer[6]. Possible explanations for the oscillations then include: as the SNR of the signal decreases with signal-time (due to acoustic attenuation) the frequency precision of the transformation decreases, and potentially, the existence of convection/turbulence in the medium. To extract a smooth measurement of the instantaneous Brillouin frequency, especially for $t \leq 1$ ns, the response is interpolated using a high order polynomial (light blue and red in Fig. S4b).

In order to measure the instantaneous sound velocity, we make the approximation that the variation in sound velocity is over an order of magnitude higher than the variation in refractive index (in water) for the frequency range spanned in Fig. S4b. This is a fair assumption if considering the thermo-optic and thermo-acoustic coefficients of water; over the temperature range $0 \leq T \leq 100$ °C, the maximum percent variation in the refractive index of water is 1%[7], the variation in sound velocity is 15%[8], and the maximum variation in Brillouin frequency is 15%. Under this assumption, the refractive index can be considered invariant resulting in a directly proportional relationship between Brillouin frequency and sound velocity (right $y$-axis in Fig. S4b).

In the manuscript, the 1D spatial vector ($z$) - of each TRBS trace in which a proximity measurement was performed - was discretised such that the $i^{\text{th}}$ element was:

$$z_{\text{c}} = z_i = \sum_{k=1}^{i} v_{\text{c}} \Delta t \tag{S.2}$$

where $v_{\text{c}}$ was measured experimentally and considered constant versus signal-time ($\Delta t = 1.25$ ps is constant). Now, using the time-varying sound velocity calculated in Fig. S4b, the spatial vector can be re-sampled as:

$$z_T = z_i = \sum_{k=1}^{i} v_k \Delta t \tag{S.3}$$

Fig. S4c demonstrates the mapping of the homogeneous (in the figure, $z_{\text{out}} = z_{\text{c}}$ from Eq. S.2) and inhomogeneous (in the figure, $z_{\text{out}} = z_T$ from Eq. S.3) spatial vectors (using the sound velocities for the red curve in Fig. S4b) compared with a homogeneous reference vector ($z_{\text{ref}} = z_{\text{c}}$), where homogeneity is represented with unity slope (dotted line). Variation in the spatial vector ($z_T$) that occurs early in the signal clock accumulates and results in a larger discrepancy between homogeneous and inhomogeneous spatial vectors (inset in Fig. S4c). The error between the two spatial vectors is then

calculated by simply taking their difference:

$$\epsilon_z(t) = z_c(t) - z_T(t) \tag{S.4}$$

This error calculation can now be applied to the three traces presented in Fig. S4a as a function of signal-time (see Fig. S4d). Despite the two NB signals slightly differing in frequency content (and frequency fluctuations), they ultimately produce similar errors on the final spatial measurement with a maximum variation of $\Delta\epsilon_z \approx 10$ nm between them. For the case of the WB signal, the positional error before the boundary transition appears to be well-behaved and in agreement with the trends exhibited by the NB cases. As with the measurement of the instantaneous Brillouin frequency, each error curve can be interpolated to smooth the response (light blue and red curves in Fig. S4d).

To estimate the maximum systematic error on the height measurement of an object, the time-position of the floor measurement is required, since this measurement will take place latest in the time-vector of the signal. For the PS-objects measured in manuscript Figs. 4c,f,i, the time-positions of the floors were measured as follows: $t_{\text{floor}} = 3.95$, 3.80, and 3.14 ns, respectively. These values are then located along the $x$-axis of Fig. S4d, and correspond with error measurements (corresponding value on the $y$-axis) of $\epsilon_z = 90$, 70, and 40 nm, respectively (using the red curve in Fig. S4d).

All proximity and height measurements in the main manuscript were performed using the weighted average sound velocity measurement from the entire time-length of the TRBS signal (measured from the position of the Brillouin spectral peak), and not using the instantaneous sound velocity across the signal time-vector. The primary reason for this decision, was to avoid discontinuities in sound velocity measurement which occur at the boundaries (as demonstrated by the dotted portion of the green curve in Fig. S4b). Due to the non-zero width of the wavelet, the edge-response of the wavelet transformation will span a non-zero distance, with the position of the boundary residing within this edge response. This edge-response occurs for both the maximum amplitude frequency (left $y$-axis of Fig. S4b) and the related instantaneous sound velocity (right $y$-axis of Fig. S4b), and results in a non-physical sound velocity measurement within the confines of the edge-response. Additionally, as the wavelet begins to cross the boundary between the couplant and object materials, the measurement of the couplant's sound velocity will become less precise since the final acoustic wavelengths in the couplant are no longer being sampled by the full-width of the wavelet. For these reasons, it is appropriate to localise boundary responses using the weighted average sound velocity of the entire time trace, and then provide an estimation of the positional error due to fluctuations in instantaneous Brillouin frequency.

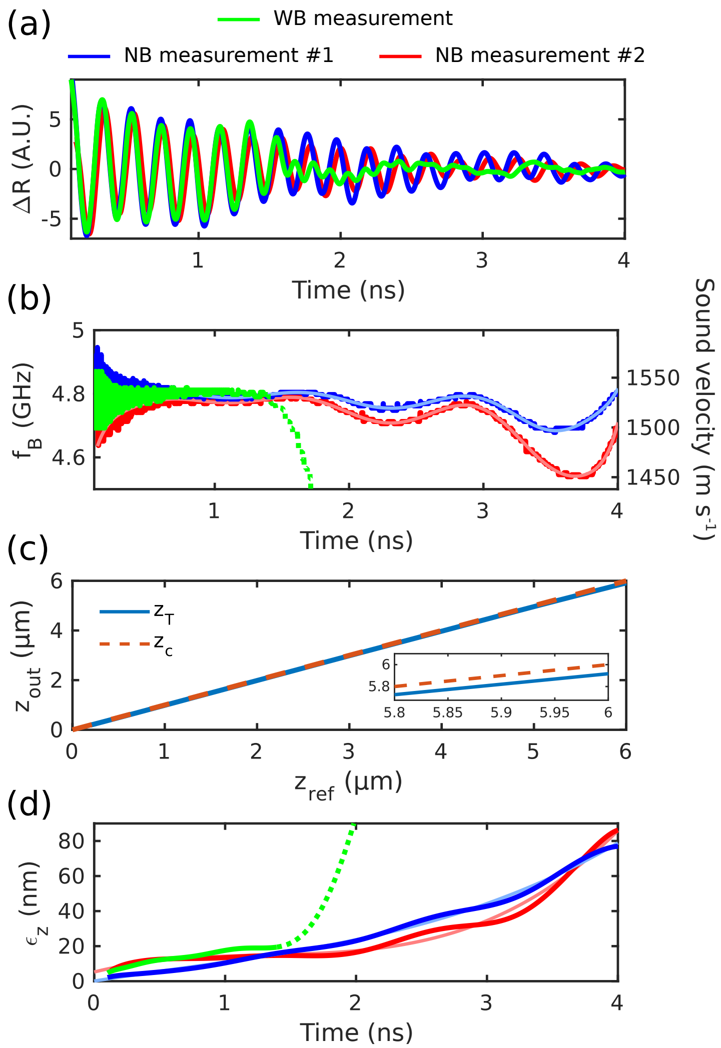


**Figure S4.** (a) Representative TRBS signals for the following cases: two separate TRBS signals without a material boundary ("NB", red and blue curves), and one TRBS signal with a material boundary ("WB", green curve) shown for comparison. (b) CWT is used to calculate the maximum amplitude frequency along the time-of-flight of the TRBS signals. The time-varying Brillouin frequency is approximated as being directly proportional to the sound velocity (right axis (b)), since this varies much more strongly than does the refractive index for changes in temperature (in water). The two NB responses display similar fluctuations in Brillouin frequency, likely due to transducer-heating and cooling in the water medium. The WB case reveals a similar response as the other two within the first 1.5 ns, followed by a steep drop-off in instantaneous Brillouin frequency (and therefore sound velocity). This is due to the wavelet sampling the lowest SNR portion of the signal (green in (a) for $t \geq 2$ ns) and results in a non-physical sound velocity measurement surrounding this fast edge-response. (c) The instantaneous sound velocity (for NB measurement #2) is used to assemble a spatial vector (blue curve, Eq. S.3, $z_{\text{out}} = z_T$). A spatial vector that was assembled using a constant sound velocity (orange dashed curve, Eq. S.2, $z_{\text{out}} = z_{\text{ref}} = z_{\text{c}}$) containing unity slope, produces a different spatial vector compared with the inhomogeneous spatial vector (as shown in the inset). (d) The systematic error that arises if one uses the homogeneous spatial vector to make a measurement is given by the difference between the homogeneous and inhomogeneous spatial vectors (Eq. S.4). Despite having subtle differences in instantaneous frequencies, all three cases produce similar trends and values for the systematic error; after $t \geq 1.5$ ns, the WB-case (green) produces large error due to the instability of the sound velocity measurement.

## References


1. Matsuda, O. & Wright, O. B. Reflection and transmission of light in multilayers perturbed by picosecond strain pulse propagation. *JOSA B* **19**, 3028–3041, DOI: 10.1364/JOSAB.19.003028 (2002).

2. Pérez-Cota, F. *et al.* Apparent attenuation by opto-acoustic defocus in phonon microscopy. *Photoacoustics* **19**, 100180, DOI: 10.1016/j.pacs.2020.100180 (2020).

3. Lanni, F., Waggoner, A. S. & Taylor, D. L. Structural organization of interphase 3T3 fibroblasts studied by total internal reflection fluorescence microscopy. *The J. Cell Biol.* **100**, 1091–1102, DOI: 10.1083/jcb.100.4.1091 (1985).

4. Arakawa, M. *et al.* Development of an ultrasound microscope combined with optical microscope for multiparametric characterization of a single cell. *IEEE Transactions on Ultrason. Ferroelectr. Freq. Control.* **62**, 1615–1622, DOI: 10.1109/TUFFC.2014.006865 (2015). Conference Name: IEEE Transactions on Ultrasonics, Ferroelectrics, and Frequency Control.

5. Norouzi, N., Bhakta, H. C. & Grover, W. H. Sorting cells by their density. *PloS one* **12**, e0180520, DOI: 10.1371/journal.pone.0180520 (2017).

6. La Cavera, S., Pérez-Cota, F., Fuentes-Domínguez, R., Smith, R. J. & Clark, M. Time resolved Brillouin fiber-spectrometer. *Opt. Express* **27**, 25064–25071, DOI: 10.1364/OE.27.025064 (2019).

7. Bashkatov, A. N. & Genina, E. A. Water refractive index in dependence on temperature and wavelength: a simple approximation. In *Saratov Fall Meeting 2002: Optical Technologies in Biophysics and Medicine IV*, vol. 5068, 393–395, DOI: 10.1117/12.518857 (International Society for Optics and Photonics, 2003).

8. Yang, F. *et al.* Picosecond ultrasonic experiments with water and its application to the measurement of nanostructures. *J. Appl. Phys.* **107**, 103537, DOI: 10.1063/1.3388283 (2010).
